# Seasonal influenza mRNA vaccine induces stronger innate and comparable or better adaptive responses than licensed inactivated vaccines

**DOI:** 10.1038/s41541-026-01492-y

**Published:** 2026-05-26

**Authors:** Erick Bermúdez-Méndez, Klara Lenart, Rodrigo Arcoverde Cerveira, Olivia Engstrand, Fredrika Hellgren, Alberto Cagigi, Sebastian Ols, Edith Jasny, Annika Reinhardt, Benjamin Petsch, Karin Loré

**Affiliations:** 1 Division of Immunology and Respiratory Medicine, Department of Medicine Solna, Karolinska Institutet and Karolinska University Hospital, Stockholm, Sweden; 2 Center for Molecular Medicine, Karolinska Institutet, Stockholm, Sweden; 3 CureVac SE, Tübingen, Germany; 4 Present Address: Laboratory of Molecular Immunology, The Rockefeller University, New York, USA; 5 Present Address: Europe Regional Office, International Vaccine Institute, Stockholm, Sweden; 6 Present Address: Department of Biochemistry and Institute for Protein Design, University of Washington, Seattle, USA; 7 Present Address: CiplaRNA GmbH, Reutlingen, Germany

**Keywords:** Diseases, Immunology, Microbiology

## Abstract

Seasonal vaccination is the most important strategy for mitigating the large disease burden caused by influenza virus. Licensed inactivated influenza virus vaccines rely on slow, egg-based production, underscoring the need for faster, more effective platforms that elicit robust immune responses. Here, we compare the immunogenicity of an unmodified mRNA-lipid nanoparticle vaccine encoding influenza hemagglutinin (HA) from A/Michigan/45/2015 (H1N1)pdm09, A/Singapore/INFIMH-16-0019/2016 (H3N2), B/Phuket/3073/2013 and B/Colorado/06/2017 with two approved, strain-matched, inactivated vaccines (Vaxigrip and Fluad) in non-human primates. The mRNA vaccine induced substantially stronger innate immune activation than Vaxigrip and Fluad, evidenced by rapid upregulation of genes involved in antiviral, antigen presentation and cell migration pathways, expansion of intermediate monocytes and increased secretion of pro-inflammatory cytokines. The mRNA vaccine elicited HA-specific antibodies against all four strains, with levels generally comparable to or exceeding those induced by Vaxigrip and Fluad, although this did not consistently translate to greater neutralizing capacity. Both the mRNA vaccine and Fluad generated higher frequencies of HA-specific memory B and T cell responses compared to Vaxigrip, with the mRNA vaccine inducing a particularly stronger response in the draining lymph nodes, potentially increasing antibody diversity and affinity. Altogether, these findings support the advancement of influenza mRNA vaccines as promising clinical candidates.

## Introduction

Yearly vaccination against predicted circulating strains is the mainstay for protection against influenza virus infections^1^. Vaxigrip (Sanofi Pasteur) and Fluad (Seqirus) are two licensed influenza vaccines widely used in immunization programs around the world. Vaxigrip is a tri- or tetravalent inactivated split-virion vaccine^2^, whereas Fluad is a tri- or tetravalent inactivated subunit vaccine formulated with the MF59 adjuvant, an oil-in-water emulsion designed to enhance immune responses, particularly in older adults^3^. The main component of both licensed vaccines consists of hemagglutinin (HA), one of the immunodominant glycoproteins on the influenza virus surface. The HAs included in the multivalent formulations derive from two influenza A subtypes (H1N1 and H3N2) and one (or two) influenza B lineages (Yamagata and/or Victoria)^4,5^.

Although the approved influenza vaccines contribute to reducing influenza-associated morbidity and mortality, their overall effectiveness varies across populations and seasons, typically ranging between 20 and 60%^6^. Suboptimal effectiveness of these vaccines largely results from the antigenic drift of circulating viruses, challenges in predicting the circulating strains, as well as induction of weak, short-lasting immunity^7–9^. Currently, several strategies to improve the potency, durability and breadth of protective responses induced by influenza vaccines are subject of active investigation, including exploring alternative vaccine platforms, such as mRNA-lipid nanoparticle (LNP) vaccines.

Protection against infection using an mRNA vaccine was first demonstrated in animal models with an influenza mRNA, marking a foundational proof of concept for the platform^10^. Subsequent biotechnological advances, including improvements in delivery systems, paved the way for mRNA-LNP vaccines to become a successful platform for combating infectious diseases, as demonstrated during the COVID-19 pandemic^11^. The early success of this technology can be attributed to its robust manufacturing process, the ease to rapidly develop vaccine candidates, flexibility to modify or switch antigens, and high level of in vivo antigen expression^12^, which may facilitate sustained germinal center formation and induction of robust humoral immunity^13,14^. In particular, the adaptability of the platform enables the simultaneous delivery of multiple influenza virus antigens, aiming to elicit protective immune responses against a broad range of influenza virus subtypes. This co-delivery can be achieved either by using separate mRNA-LNPs, each encoding a distinct antigen^15–17^, by combining distinct mRNAs into a single mixture before LNP encapsulation, or by designing chimeric constructs encoding multiple influenza virus antigens within a single mRNA molecule^18^.

Here, we performed a detailed characterization of the innate and adaptive immune responses induced by prime-boost vaccination with a tetravalent HA-coding unmodified mRNA vaccine candidate, prepared by mixing the four mRNAs before formulating them into a single LNP preparation, compared to Vaxigrip and Fluad in non-human primates. The strong innate immune activation and similar or higher HA-specific IgG, memory B cell and T cell responses triggered by the mRNA vaccine compared to the licensed vaccines highlight the mRNA’s promise as next-generation seasonal influenza vaccines.

## Results

### mRNA vaccine induces an overall stronger innate immune response than the inactivated vaccines

**Fig. 1:**
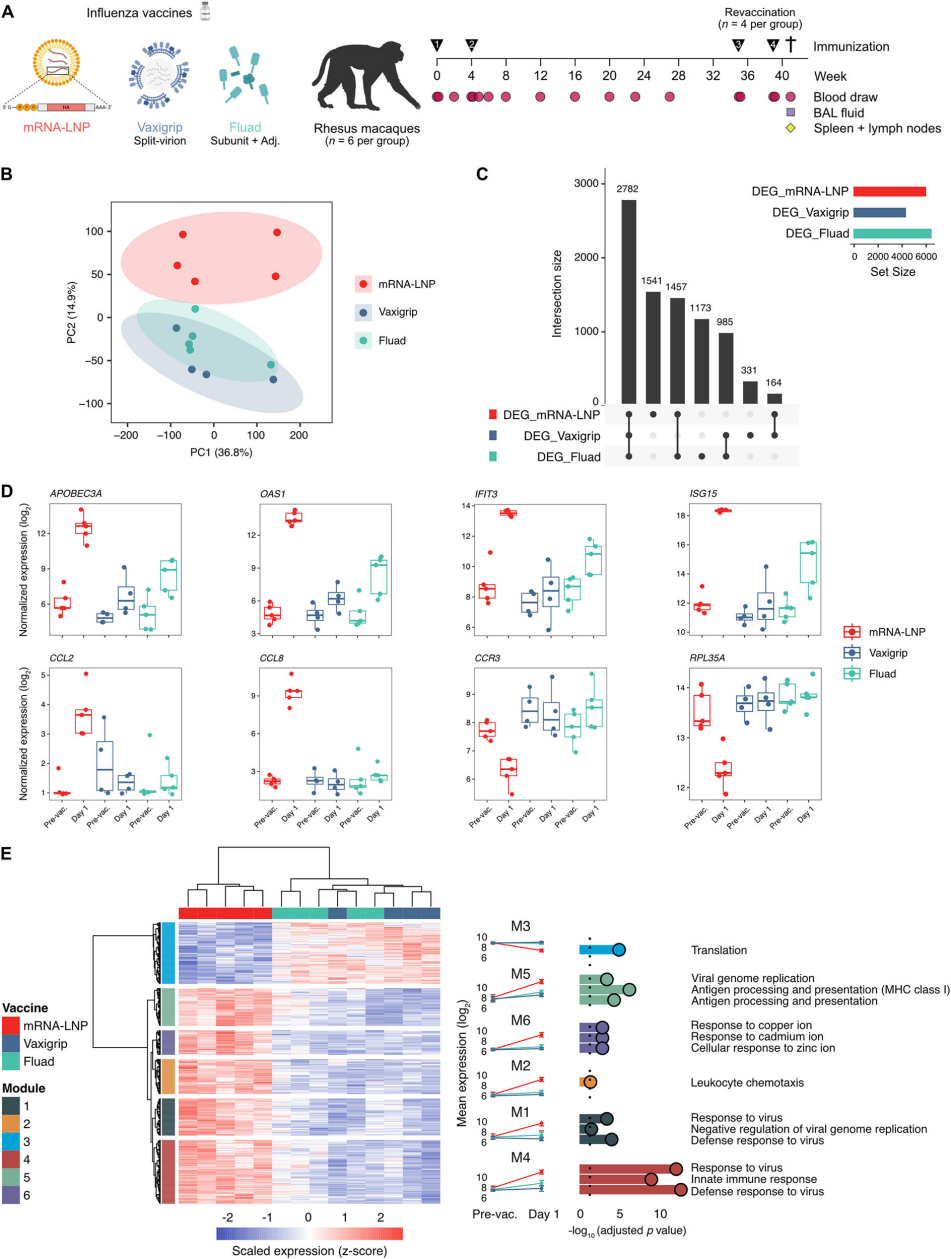
Study design and transcriptomic alterations induced after vaccination. **A** Study design. Eighteen rhesus macaques (*n* = 6 per group) were intramuscularly immunized at weeks 0 and 4, and revaccinated (*n* = 4 per group) at weeks 35 and 39 with either an unmodified mRNA-lipid nanoparticle (LNP) influenza vaccine, an inactivated split-virion vaccine (Vaxigrip) or an adjuvanted inactivated subunit vaccine (Fluad). Blood, bronchoalveolar lavage (BAL) fluid, spleen and lymph node samples were collected at various time points for a broad range of immunological assessments. Illustration was partially created with BioRender.com. **B** Principal component analysis of blood samples based on their differential transcriptomic profile before and one day after the first immunization. Dots represent individual animals (*n* = 4-5 per group). **C** Upset plot representing the numbers of total, shared (intersection) and unique differentially expressed genes (before and one day after the first immunization comparison) among the different groups. Genes with an absolute log_2_ fold change of 1 and a *q*-value < 0.05 were considered significantly differentially expressed. **D** Expression profiles of 8 selected genes before and one day after the first immunization. Dots represent normalized gene counts (log_2_ scale) of individual animals, boxes represent the interquartile range (Q1-Q3) and horizontal lines show the mean per group. **E** Functional transcriptomic analysis. Clustered heat map of differentially expressed genes among the three vaccine groups, split into various functional modules (M1-M6) displaying similar expression behavior (left). Mean gene expression (log_2_ scale) for each module before and one day after the first immunization (middle). Top gene ontology (GO) database pathways significantly enriched (adjusted *p*-value < 0.05) within each gene module (right).

To benchmark a candidate mRNA influenza vaccine against two licensed inactivated vaccines (Vaxigrip and Fluad), we conducted a head-to-head comparison of their safety and immunogenicity profiles in non-human primates. This model enabled frequent sampling and collection of multiple tissues to gain mechanistic insights into the immune responses. Eighteen rhesus macaques (*n* = 6 per vaccine group) received a prime dose (day 0) of either the mRNA vaccine encoding HA of four different strains (A/Michigan/45/2015 [H1N1]pdm09, A/Singapore/INFIMH-16-0019/2016 [H3N2], B/Phuket/3073/2013 and B/Colorado/06/2017; 40 µg of mRNA per strain) or the full human dose of Vaxigrip (containing the same four strains) or Fluad (containing the same strains except B/Phuket/3073/2013). This was followed by a second (booster) dose with the respective vaccine four weeks after prime (week 4). Thirty-five weeks (eight months) after the prime dose, 12 of the 18 macaques (*n* = 4 per vaccine group) received a third (week 35) and a fourth (week 39) dose within a four-week interval (Fig. 1A).

We chose a high 160 µg dose and a multiple immunization regimen because the macaques were influenza-naive at the start of the study. This approach allowed us to investigate the effects of repeated vaccination with the mRNA platform, including the potential for boosting adaptive responses or the risk of exaggerating innate immune sensing following multiple immunizations, which are critical considerations for the long-term application of mRNA vaccines. However, it is important to note that the mRNA dose used in our study is considerably higher than the dose used in recent clinical trials (e.g., 50 µg of Moderna’s mRNA-1010)^19,20^. All immunizations were administered intramuscularly. Blood samples were collected at multiple timepoints throughout the study period. Additionally, bronchoalveolar lavage (BAL) fluid, spleen and lymph node samples were collected at week 41 (study end).

We first investigated the early innate immune responses in peripheral blood one day after vaccine administration by combining RNA microarrays to assess vaccine-induced changes in gene expression, flow cytometry for phenotyping of cells and Luminex assays for assessment of plasma cytokines. Transcriptomic analysis of whole blood before and one day after the prime dose revealed that all three vaccines induced extensive gene expression changes (differential expression defined as an absolute log_2_ fold change of 1 and a *q-*value < 0.05; Supplementary Fig. 1A and Supplementary Data 1-3). Samples from the mRNA group formed a distinct cluster in a principal component analysis, indicating clear differences in gene expression signatures induced by the mRNA vaccine compared to Vaxigrip and Fluad (Fig. 1B).

Among the differentially expressed genes, a large set of 2,782 genes was commonly found across samples from all groups (Fig. 1C and Supplementary Data 4). Notably, gene expression changes induced by the mRNA vaccine were typically stronger in magnitude (Supplementary Fig. 1A). For instance, within the shared set of genes, *APOBEC3A* and *OAS1* were substantially more upregulated after immunization with the mRNA vaccine than with Vaxigrip and Fluad (Fig. 1D). Similarly, genes differentially expressed in the mRNA and Fluad groups (e.g., *IFIT3* and *ISG15*) also showed markedly higher expression levels after vaccination in the mRNA group relative to the other vaccines. Interestingly, some genes were exclusively upregulated (e.g., *CCL2* and *CCL8*) or downregulated (*CCR3* and *RPL35A*) in the mRNA group (Fig. 1D). A functional analysis of the differentially expressed genes highlighted that modules relating to antiviral responses, antigen processing and presentation, as well as chemotaxis and cell migration were among the most important significantly upregulated modules in the mRNA group compared to the inactivated vaccines groups (Fig. 1E and Supplementary Fig. 1B). In contrast, genes involved in protein translation pathways were downregulated solely in the mRNA group (Fig. 1E), consistent with the activation of antiviral response mechanisms induced by mRNA administration.

**Fig. 2:**
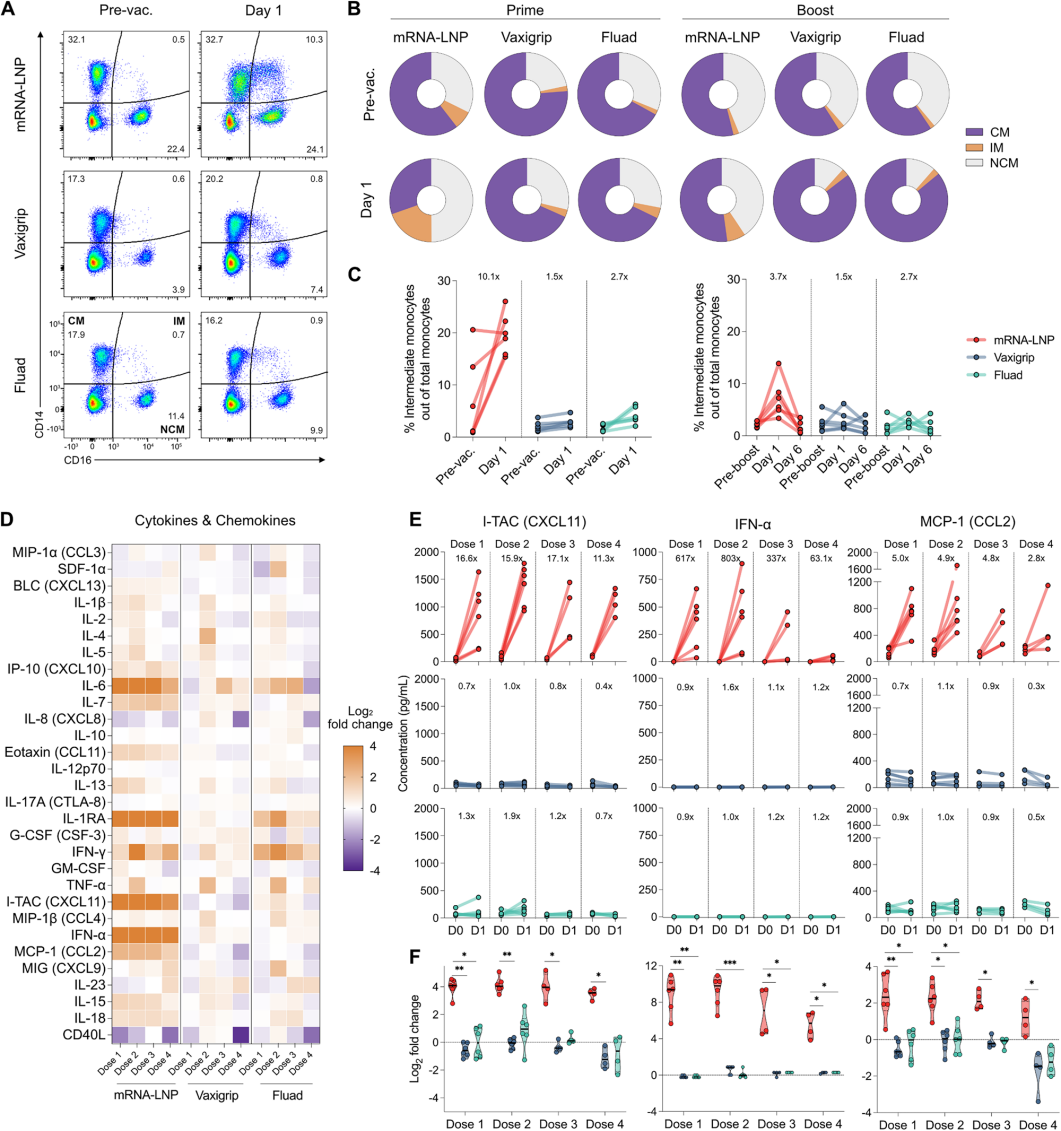
Unmodified mRNA-LNP vaccine induces a stronger innate immune response than the inactivated vaccines. **A** Representative innate immunophenotyping flow cytometry plots of peripheral blood mononuclear cells (PBMCs) for quantification of classical (CD14^+^CD16^-^), intermediate (CD14^+^CD16^+^) and non-classical monocytes (CD14^-^CD16^+^) before and one day after the first immunization. CM, classical monocytes; IM, intermediate monocytes; NCM, non-classical monocytes. Gating strategy for the identification of the different monocyte populations is shown in Supplementary Fig. 2. **B** Mean relative frequencies of classical, intermediate and non-classical monocytes before and one day after the first (left) and second (right) immunizations per vaccine group (*n* = 6 rhesus macaques per group). **C** Fluctuations in the relative frequency of intermediate monocytes (CD14^+^CD16^+^) among total monocytes before and one day after the first (left) and second (right) immunizations. Additionally, data at six days after the second immunization are shown. Dots linked by a straight line show paired data per individual animal (*n* = 6 per group). Mean fold changes per group are shown at the top. **D** Heat map of mean changes in plasma cytokine and chemokine levels (log_2_ fold changes) before and one day after each of the four immunizations for each vaccine group measured in a Luminex assay (*n* = 6 per group for the first and second doses; *n* = 4 per group for the third and fourth doses). Measurements of cytokines and chemokines at undetectable levels or below the lower limit of quantification were set to the lower limit of quantification for calculation of fold changes and plotting. Log_2_ fold changes lower than -4 or higher than +4 were set to -4 and +4, respectively, for visualization purposes. **E** Fluctuations in the concentration of selected cytokines and chemokines in plasma before and one day after each of the four immunizations. Dots linked by a straight line show paired data per individual animal (*n* = 6 per group for the first and second doses; *n* = 4 per group for the third and fourth doses). Mean fold changes per dose for each group are shown at the top of each plot. **F** Fold changes (log_2_ scale) in the concentration of selected cytokines and chemokines in plasma before and one day after each of the four immunizations. Dots represent fold changes of individual animals (*n* = 6 per group for the first and second doses; *n* = 4 per group for the third and fourth doses) and horizontal lines show the group median. Significant statistical difference between the group medians was determined with a Kruskal-Wallis test with Dunn’s multiple comparisons correction. *p < 0.05, **p < 0.01, ***p < 0.001.

We and others have previously shown that the rapid expansion of circulating CD14^+^CD16^+^ intermediate monocytes is a hallmark feature of robust innate immune activation upon immunization^21–24^. The transient expansion of intermediate monocytes after the prime dose in the Vaxigrip and Fluad groups was clear but moderate (1.5x and 2.7x, respectively), whereas there was a significantly stronger expansion of intermediate monocytes in the mRNA group (10.1x) (Fig. 2A-C and Supplementary Figs. 2-3). Similar trends were observed following the booster dose. At day 6 post-vaccination, circulating intermediate monocytes returned to near baseline levels in all the groups (Fig. 2C and Supplementary Fig. 3).

Besides the transient expansion of intermediate monocytes, plasma levels of pro-inflammatory IFN-γ, interleukin 6 (IL-6) and IL-7 increased following vaccination with either of the three vaccines (Fig. 2D). Notably, a sharp increase in additional pro-inflammatory cytokines and chemokines including IFN-ɑ, IFN-inducible T cell alpha chemoattractant (I-TAC), monocyte chemoattractant protein 1 (MCP-1), IL-1β, IFN-γ-induced protein 10 (IP-10), eotaxin (CCL11) and IL-15 was observed only in the mRNA group (Fig. 2D-F). Despite inducing a strong systemic innate immune activation after each immunization, the mRNA vaccine showed generally comparable results to the licensed vaccines in safety parameters such as body temperature, weight, hematological profile and clinical chemistry markers (Supplementary Figs. 4-6). Together, these data show that the mRNA vaccine elicited a greater innate immune response than the two licensed inactivated vaccines Vaxigrip and Fluad.

### Vaccine-elicited antibody responses are heterogeneous and strain-dependent regardless of the vaccine type

**Fig. 3:**
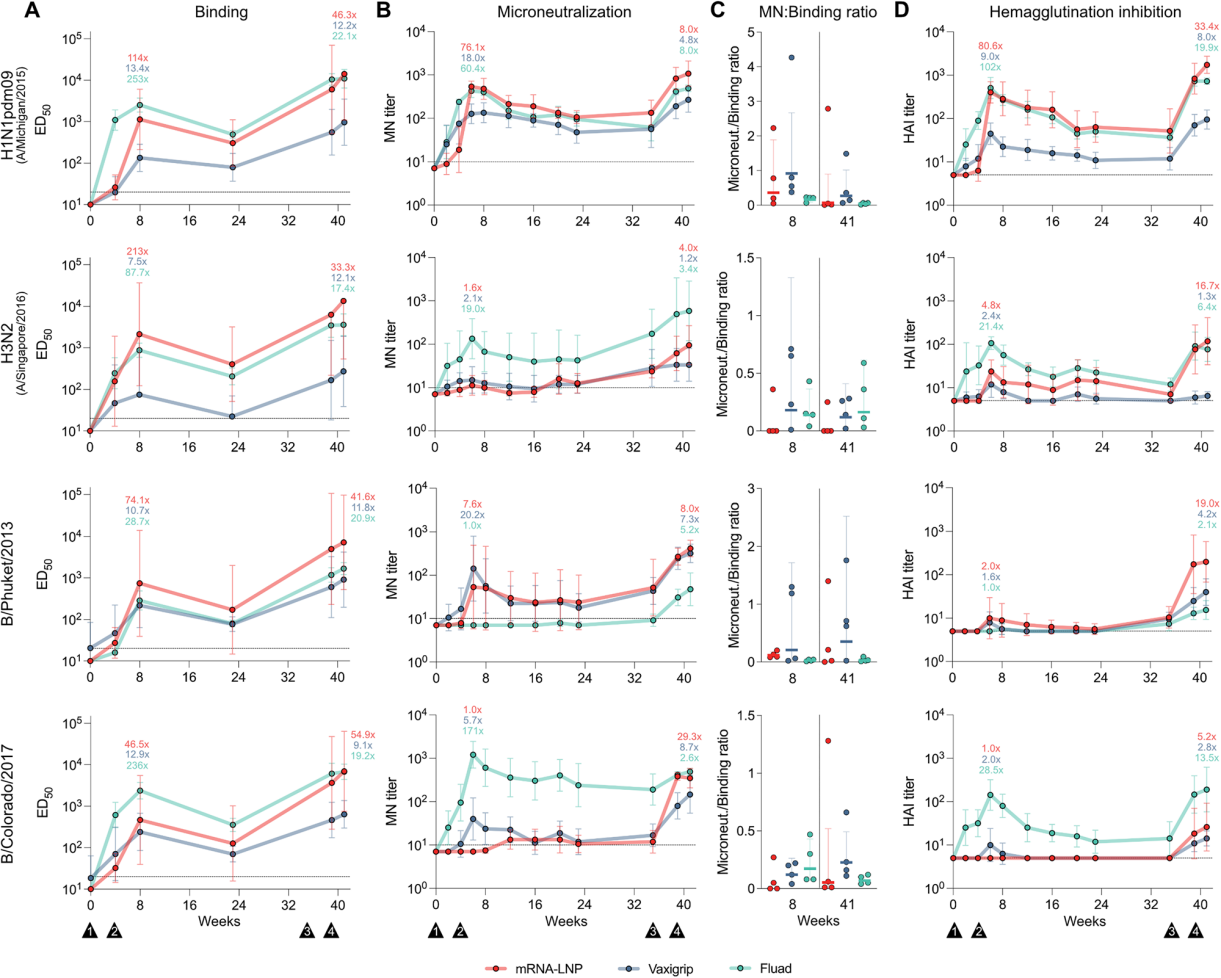
Vaccine-elicited antibody responses. **A** Vaccine-induced HA-specific plasma IgG binding titers against the indicated influenza strains determined by ELISA. Graphs show geometric mean half-maximal effective dilutions (ED_50_) with geometric standard deviations (SD) at each timepoint (*n* = 4 per group). Geometric mean fold changes at week 8 relative to baseline (week 0) and at week 41 relative to week 23 are shown at the top of each plot. **B** Serum microneutralizing (MN) antibody titers against the indicated influenza strains. Graphs show geometric mean MN titers with geometric SD at each timepoint (*n* = 6 per group until week 23 and *n* = 4 per group for weeks 35-41). Geometric mean fold changes at week 6 relative to baseline (week 0) and at week 41 relative to week 35 are shown at the top of each plot. MN titers are reported as the reciprocal of the highest dilution where no cytopathic effect was observed. **C** Microneutralization-to-binding ratios against the indicated influenza strains at weeks 8 and 41 (four weeks after the second immunization and 2 weeks after the fourth immunization, respectively). Dots represent ratios of individual animals (*n* = 4 per group). Horizontal lines indicate geometric means per group and error bars show geometric SDs. **D** Serum hemagglutination inhibition (HAI) titers in response to the indicated influenza strains. Graphs show geometric mean HAI titers with geometric SD at each time point (*n* = 6 per group until week 23 and *n* = 4 per group for weeks 35-41). Geometric mean fold changes at week 6 relative to baseline (week 0) and at week 41 relative to week 35 are shown at the top of each plot. HAI titers are reported as the reciprocal of the last serum dilution that resulted in no agglutination. Dashed lines indicate the limits of detection (ED_50_ = 20, MN = 10 and HAI = 5, for the ELISA, MN assay and HAI assay, respectively). Measurements below the lower limit of detection were set to ED_50_ = 10, MN = 7.07 and HAI = 5, for the ELISA, MN assay and HAI assay, respectively. Black triangles at the bottom indicate the timing of the four immunizations.

To investigate the humoral adaptive immune responses after vaccination, we measured the HA-specific IgG levels, neutralization titers and hemagglutination inhibition (HAI) titers against the four matching strains in the vaccines (A/Michigan/45/2015 [H1N1]pdm09, A/Singapore/INFIMH-16-0019/2016 [H3N2], B/Phuket/3073/2013 and B/Colorado/06/2017). Antibodies against the different HAs elicited by all three vaccines were detectable throughout the study and generally followed a similar trend over time. HA-specific antibodies increased by four weeks post-vaccination and reached a first peak (7.5x increase or higher) four weeks after the booster dose (week 8). By 23 weeks post-vaccination, HA-specific antibody levels had declined at a roughly similar rate in all the groups. However, revaccination at study week 35 further amplified the HA-specific response, reaching higher levels than the first peak response (Fig. 3A). Although the three vaccines induced a similar kinetics in antibody responses, notable differences were observed in terms of magnitude and depending on the specific HA strain. In general, Vaxigrip induced lower anti-HA antibody levels than the mRNA and Fluad vaccines for all four strains. HA-specific antibodies elicited by the mRNA vaccine were higher than those elicited by Fluad for the H3N2 (A/Singapore/INFIMH-16-0019/2016) and B/Phuket/3073/2013 strains, but reached similar levels for the H1N1pdm09 (A/Michigan/45/2015) and B/Colorado/06/2017 strains (Fig. 3A). The detection of HA-specific antibodies against the B/Phuket/3073/2013 strain in the Fluad group is likely due to cross-reactivity, as this strain is not included in the vaccine.

Interestingly, the high levels of HA-specific antibodies elicited by the mRNA vaccine did not consistently correlate with better virus neutralization capacity against the four influenza strains. For H1N1pdm09 (A/Michigan/45/2015) and B/Phuket/3073/2013, the neutralizing titers induced by the mRNA vaccine were similar to those of the licensed comparators. However, for H3N2 (A/Singapore/INFIMH-16-0019/2016) and B/Colorado/06/2017, neutralizing titers were lower than those observed with the MF59-adjuvanted Fluad (Fig. 3B). High HA-specific antibody levels elicited by Fluad did translate to high titers of neutralizing antibodies against the three strains included in that vaccine.

While Vaxigrip elicited comparable or lower neutralizing titers than the other vaccines, its neutralization-to-binding antibody ratios suggest it may induce antibodies of relatively high functional quality. The distinct patterns of virus neutralization capacity in relation to the magnitude of HA-specific binding antibodies are reflected in the ratios presented in Fig. 3C. As expected, Fluad failed to induce neutralizing antibodies against the B/Phuket/3073/2013 strain not included in the formulation. Overall, the neutralizing titers elicited by the mRNA vaccine were comparable or slightly higher than those observed with Vaxigrip, while the MF59-adjuvanted Fluad generally induced stronger neutralizing responses against matched strains (Fig. 3B). These findings demonstrate the robust antibody response induced by the mRNA vaccine, while also reflecting the complexity of correlating antibody levels with neutralizing capacity across different influenza strains.

HAI titers against the H1N1pdm09 (A/Michigan/45/2015) and H3N2 (A/Singapore/INFIMH-16-0019/2016) influenza A strains exhibited similar patterns upon immunization in all the groups, with titers peaking two to four weeks after the second and fourth immunization. Compared to Vaxigrip, the mRNA and Fluad vaccines both induced higher HAI responses (Fig. 3D). HAI titers against both influenza B strains were rather low in magnitude. However, after revaccination (weeks 39 and 41), the mRNA vaccine induced a higher HAI response against the B/Phuket/3073/2013 strain compared to Vaxigrip and Fluad. For the B/Colorado/06/2017 strain, Fluad induced high HAI titers already after the prime-boost immunization, with sustained HAI titers at higher levels than in the comparator groups (Fig. 3D). Overall, the results demonstrate that all three vaccines elicited HA-specific neutralizing antibodies, but the functionality of the antibodies varied across the different strains.

### Induction of circulating HA-specific memory B and T cell responses

**Fig. 4:**
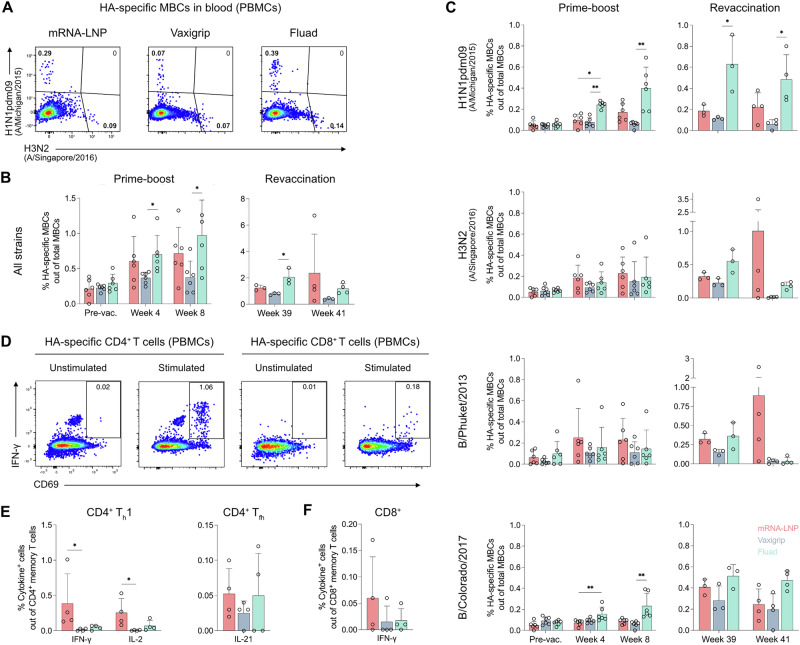
Induction of circulating HA-specific memory B cell and T cell responses. **A** Representative flow cytometry plots depicting the detection and quantification of HA-specific memory B cells (MBCs) in blood at week 8. Gating strategy for the identification of HA-specific MBCs is shown in Supplementary Fig. 7. **B** Relative frequency of total HA-specific MBCs (cumulative across all strains) among total MBCs in blood at baseline and weeks 4, 8, 39 and 41. **C** Strain-specific relative frequencies of HA-specific MBCs among total MBCs in blood at baseline and weeks 4, 8, 39 and 41. **D** Representative flow cytometry plots depicting the detection and quantification of IFN-γ-producing CD4^+^ and CD8^+^ memory T cells in blood at week 41 (two weeks after the fourth immunization) upon in vitro re-stimulation with a mix of overlapping HA peptides from the four influenza strains. Gating strategy preceding the identification of HA-specific memory T cells is shown in Supplementary Fig. 8. **E**, **F** Relative frequency of HA-specific cytokine-producing CD4^+^ and CD8^+^ T cells among total CD4^+^ and CD8^+^ memory T cells, respectively, in blood at week 41 (two weeks after the fourth immunization). Results were background subtracted based on values from unstimulated cells. Dots represent data for individual animals (*n* = 3-6 per group). Bars show the group means and error bars indicate the SD. Significant statistical difference between the group medians was determined with a Kruskal-Wallis test with Dunn’s multiple comparisons correction. *p < 0.05, **p < 0.01.

To evaluate induction of peripheral memory B cell responses following immunization, we performed staining with HA probes and flow cytometry analysis on blood samples (Fig. 4A and Supplementary Fig. 7). Circulating HA-specific memory B cells were detected in all vaccine groups after the prime-boost regimen (Fig. 4B), although their levels varied across the different HA antigens (Fig. 4C). Overall, the Fluad and mRNA vaccines induced a more pronounced memory B cell response than that induced by Vaxigrip (Fig. 4B). Fluad predominantly generated HA-specific memory B cells directed against the H1N1pdm09 (A/Michigan/45/2015) and B/Colorado/06/2017 strains, whereas vaccination with the mRNA vaccine elicited the highest frequencies of HA-specific memory B cells targeting the H3N2 (A/Singapore/INFIMH-16-0019/2016) and B/Phuket/3073/2013 strains. Similar response patterns were observed upon revaccination (third and fourth doses). These later doses led to slightly higher frequencies of HA-specific memory B cells compared to the first two doses, suggesting that revaccination effectively expanded HA-specific B cell clones, particularly in the mRNA and Fluad groups (Fig. 4B-C). Collectively, these results show that all three vaccines readily generated a pool of HA-specific memory B cells in the circulation, with the mRNA and Fluad vaccines showing greater magnitude and reactivity towards distinct influenza strains.

In addition to memory B cells, we assessed HA-specific memory T cell responses in peripheral blood mononuclear cells (PBMCs) collected two weeks after the final immunization (week 41). Cells were stimulated with a mix of overlapping HA peptides from the four influenza strains, and the production of intracellular cytokines was measured by flow cytometry (Fig. 4D and Supplementary Fig. 8). The mRNA vaccine elicited a strong T_h_1-type response, showing a clear trend toward higher frequencies of IFN-γ and IL-2-producing CD4^+^ memory T cells compared to Fluad and Vaxigrip (Fig. 4E). In contrast, IL-13 and IL-17-producing T cells were either undetectable or present at very low levels (Supplementary Fig. 9). HA-specific CD8^+^ memory T cell responses followed a similar pattern as their CD4^+^ memory T cell counterparts. Although their overall magnitude was low, the mRNA vaccine induced higher CD8^+^ memory T cell responses than the comparator vaccines, albeit without reaching statistical significance (Fig. 4F).

### Vaccine-induced HA-specific memory B and T cell responses in lymphoid tissues

**Fig. 5:**
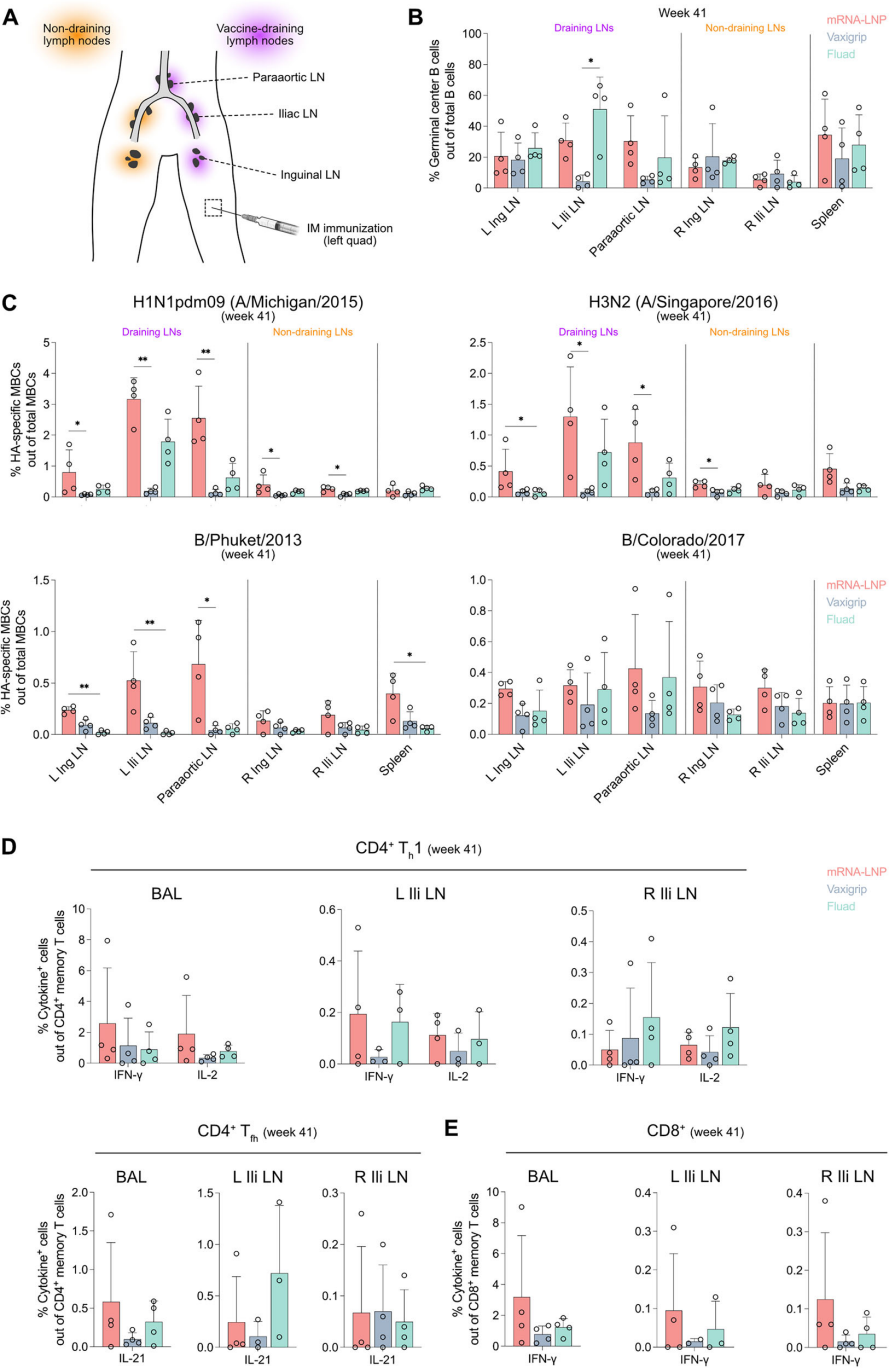
Vaccine-induced HA-specific memory B and T cell responses in lymphoid tissues. **A** Schematic illustration depicting the anatomical location of the site of immunization, vaccine-draining and non-draining lymph nodes. Lymph node samples were collected at study end (week 41) from macaques that received four immunizations. LN, lymph node; IM, intramuscular. The syringe icon was retrieved from BioRender.com. **B** Relative frequency of germinal center B cells among total B cells in lymph nodes and spleen at week 41 (two weeks after the fourth immunization). L, left; R, right; Ing, inguinal; Ili, iliac. Gating strategy for the identification of germinal center B cells is shown in Supplementary Fig. 10. **C** Relative frequency of HA-specific MBCs among total MBCs in lymph nodes and spleen at week 41. **D**, **E** Relative frequency of HA-specific cytokine-producing CD4^+^ and CD8^+^ T cells among total CD4^+^ and CD8^+^ memory T cells, respectively, in BAL fluid and lymph nodes at week 41. Dots represent data for individual animals (*n* = 2-4 per group). Bars show the group means and error bars indicate the SD. Significant statistical difference between the group medians was determined with a Kruskal-Wallis test with Dunn’s multiple comparisons correction. *p < 0.05, **p < 0.01.

We have previously shown that, after injection, both unmodified and modified mRNA vaccines, as well as protein-based vaccines, disseminate in a restricted manner to vaccine-draining lymph nodes. There, they locally induce germinal center formation and T cell responses^25–29^. To investigate whether these immune responses elicited locally differed between the vaccines, we analyzed cellular responses in lymphoid tissues, including the spleen, vaccine-draining lymph nodes and non-draining lymph nodes at week 41 (two weeks after the fourth immunization) in 12 of the 16 non-human primates (Fig. 5A). As observed previously^27,28^, germinal center B cells were mainly detected in the vaccine-draining iliac lymph nodes over inguinal lymph nodes after intramuscular immunization. Germinal center B cell frequencies were generally higher in the vaccine-draining lymph nodes and spleen of the mRNA and Fluad groups compared to the Vaxigrip group (Fig. 5B and Supplementary Fig. 10). The mRNA vaccine induced the highest and most robust HA-specific memory B cell responses in the vaccine-draining lymph nodes across all four influenza strains (Fig. 5C). While Fluad did elicit moderate responses against the three matching strains (except against the B/Phuket/3073/2013 strain, absent from its formulation), Vaxigrip only induced detectable HA-specific memory B cells against the two influenza B strains (Fig. 5C).

As expected, frequencies of HA-specific memory B cells were generally lower in non-draining lymph nodes compared to vaccine-draining lymph nodes. However, some responses were still detectable in the non-draining lymph nodes, likely due to the circulation of memory B cells. The mRNA vaccine tended to produce slightly higher responses compared to the other two vaccines, which could potentially be related to more extended systemic dissemination, although this was not formally assessed in this study. In the spleen, the number of HA-specific memory B cells were again highest in the mRNA group, at least against the H3N2 (A/Singapore/INFIMH-16-0019/2016) and B/Phuket/3073/2013 strains (Fig. 5C). Together, these findings demonstrate that all three vaccines readily induced HA-specific memory B cells in lymphoid tissues, with the mRNA vaccine showing the most robust responses.

Finally, we characterized tissue-associated T cell responses by analyzing HA-specific memory T cells derived from BAL fluid and lymph nodes (Fig. 5D-E and Supplementary Fig. 9). Although the differences were not statistically significant, the mRNA and Fluad groups generally exhibited more pronounced detectable responses compared to Vaxigrip. The mRNA vaccine group exhibited a trend toward higher frequencies of IFN-γ-producing CD4^+^ and CD8^+^ memory T cells in BAL fluid, indicating a stronger induction of cellular immune responses in the respiratory mucosa compared to Fluad and Vaxigrip (Fig. 5D-E). CD4^+^ and CD8^+^ T cell responses in the lymph nodes were robust following mRNA vaccination, demonstrating effective activation of cellular immunity. Fluad induced similar or more pronounced T cell responses in lymph nodes than the comparator groups (Fig. 5D). These data suggest that the mRNA vaccine may be more effective at inducing HA-specific tissue-associated T cells in the respiratory mucosa, potentially due to its robust innate immune activation and/or prolonged antigen expression following immunization, underscoring its advantages for next-generation influenza vaccination strategies.

## Discussion

Annual influenza vaccination is a widely recommended public health measure. Several licensed influenza vaccines are available for human use, including inactivated, live-attenuated and recombinant protein formulations^30^. One of the main challenges in achieving high clinical effectiveness lies in accurately predicting and matching the vaccine strains to those circulating in a given influenza season^4,9^. The WHO Global Influenza Surveillance and Response System publishes the recommended strains to be included in the vaccines usually around February and September each year^31^. Given the rapid production timeline of mRNA vaccines, this platform could offer a significant advantage over traditional approaches by enabling more timely selection of influenza strains, which may improve antigenic match and ultimately overall vaccine effectiveness.

Unlike the time-intensive traditional manufacturing that requires inoculation of eggs with the few chosen influenza virus strains, the mRNA platform avoids egg-adapted mutations to certain viral epitopes that may compromise vaccine immunogenicity^32–35^. While Fluad is primarily recommended for older adults and immunocompromised individuals due to its adjuvant properties, and Vaxigrip is typically administered to the general population, an mRNA influenza vaccine could represent a promising platform with wider applicability across age and risk groups. Additionally, the mRNA technology allows for the inclusion of a broader array of antigens from multiple viral strains, potentially increasing the breadth of vaccine-induced antibody responses. Recent preclinical studies have demonstrated that multivalent mRNA vaccines targeting various influenza A and B strains, and even all 20 known subtypes of influenza virus, can induce cross-reactive and subtype-specific antibodies that mediate protection against viral challenge in both mice and ferrets^16–18,36^. Several of these candidates are now advancing through clinical trials^19,37^ (reviewed by Pardi & Krammer^11^), highlighting the potential of mRNA-based approaches to improve influenza prevention.

This study aimed to comprehensively characterize the immune responses induced by a candidate HA-coding unmodified mRNA influenza vaccine, benchmarked to the licensed inactivated vaccines Vaxigrip and Fluad in non-human primates. We observed that immunization with the mRNA vaccine induced a stronger type I IFN-dominated response early after immunization compared to traditional licensed vaccines targeting the same pathogen. We have previously demonstrated a similar pattern comparing the traditional licensed inactivated virus vaccine against rabies to an mRNA vaccine encoding the rabies virus glycoprotein^22^. This strong and early type I IFN response following mRNA vaccination likely contributes to the strong T_h_1-polarized CD4^+^ T cell memory response also observed in this and several other mRNA vaccine studies^38,39^. The T_h_1 polarization could, in turn, amplify downstream B cell responses, including the generation of plasmablasts and memory B cells, as well as elevating the production of antibodies^28,40,41^.

The pronounced innate response following mRNA vaccination was marked by a transient increase in CD14^+^CD16^+^ intermediate monocytes, coinciding with elevated levels of the monocyte-attracting chemokines CCL2 (MCP-1) and CCL8 (MCP-2). These findings suggest enhanced monocyte differentiation and egress from the bone marrow into the circulation. Beyond their implication in antigen presentation to CD4^+^ T cells^42^ and in directly supporting plasmablast differentiation^43^, the expansion of this specific monocyte subset accompanied by a rise in pro-inflammatory cytokines/chemokines has been linked to broader neutralizing antibody responses after mRNA vaccination^21^. While the exact mechanisms remain to be fully elucidated, it is hypothesized that this innate activation provides critical co-stimulatory signals that improve the quality and breadth of the neutralizing response. Importantly, the safety parameters, including body weight, temperature, hematological profile and clinical chemistry markers, were only mildly affected after immunization with the mRNA vaccine candidate and fell within the normal range observed after immunization with other vaccines^44^. This capacity to trigger a robust early innate response represents a key advantage of the mRNA platform and could help explain why mRNA vaccines have proven effective in elderly and immunocompromised individuals during the SARS-CoV-2 pandemic^45–50^. These populations, particularly vulnerable to influenza infection and prioritized for seasonal vaccination, may benefit from the strong immunostimulatory properties of mRNA-based vaccines.

Other factors that may underlie the distinct immune profile induced by the mRNA vaccine are its capacity for prolonged antigen expression and systemic dissemination of the mRNA and its encoded antigen^14,28,51,52^. These features of the mRNA vaccine could account for the more effective stimulation of mucosal tissue-associated memory T cells in our study. The induction of tissue-associated memory T cells in the respiratory tract may enable the immune system to rapidly detect and respond to influenza virus at the primary site of infection. These cells can exert effector functions immediately upon pathogen encounter, potentially helping to contain and clear the infection before it spreads^53–55^. While antibodies primarily target the variable regions of HA, T cells can recognize more conserved epitopes, including those in the HA stem, which may contribute to cross-strain protection, even when antigenic drift occurs. This is particularly relevant for influenza virus, which mutates rapidly and can evade antibody-mediated immunity. In addition, the extended antigen availability might improve the quality of germinal center reactions, potentially supporting the development of more robust and longer-lasting responses. Thus, vaccines that elicit both strong antibody and T cell responses, such as mRNA vaccines, may provide broader and more durable protection.

While the mRNA vaccine induced a more prominent innate immune response one day post-immunization compared to Vaxigrip and Fluad, all three vaccines generated appreciable HA-specific memory B cell responses, as well as virus-neutralizing and HAI antibody titers. HA-binding antibody levels across all groups ranged from moderate to high depending on the influenza strain. For the H3N2 (A/Singapore/INFIMH-16-0019/2016) and B/Colorado/06/2017 strains, neutralizing antibody titers elicited by the mRNA vaccine were lower than those observed with Fluad, potentially reflecting qualitative differences in the immune responses generated by each platform. These differences, as indicated by microneutralization-to-binding ratios, highlight the complexity of vaccine-induced immunity. Further studies are warranted to determine how these immune response profiles may influence the diversity and functionality of HA-specific memory B cells and antibodies.

The mRNA vaccine encodes only HAs, whereas licensed influenza vaccines, such as Fluad and Vaxigrip, contain both HA and neuraminidase (NA). Notably, Fluad has been reported to have the highest NA content among several tested influenza vaccines^56^. As a result, licensed vaccines are capable of inducing anti-NA immune responses in addition to anti-HA responses. While HA is the principal antigen measured in microneutralization assays, NA may also contribute to virus neutralization, particularly in certain H3N2 strains where NA can mediate binding to sialic acid residues, thereby mimicking HA activity in these assays^57^. Therefore, interpretation of microneutralization assay results should consider the specific viral strain, assay format and the potential contribution of NA-mediated effects. The use of NA inhibitors may be necessary to accurately distinguish HA-specific neutralizing activity.

Expanding mRNA vaccine designs to include NA antigens offers significant clinical potential. The inclusion of NA mRNA has been shown to enhance cross-reactive immunity and significantly reduce viral shedding in the upper respiratory tract^58^, a key area where HA-only vaccines often underperform. Recent phase 1/2 clinical data suggest that dual HA-NA nucleoside-modified mRNA vaccines are well-tolerated and can elicit robust NA-specific antibodies without increasing reactogenicity^59^. However, this approach introduces several technical and immunological hurdles. Achieving balanced co-expression remains a challenge, often requiring complex multicistronic designs or self-amplifying mRNA constructs to ensure both antigens are produced in sufficient quantities^58,60^. Furthermore, there is evidence of potential antigen competition, where simultaneous expression of HA and NA may dampen individual CD8^+^ T cell responses compared to monocistronic delivery^58^. Ensuring the correct tetrameric folding of the NA protein is also critical for presenting functional epitopes, requiring careful sequence optimization. Despite these challenges, incorporating NA into mRNA formulations represents a promising strategy for developing vaccines with improved breadth and potential transmission-reducing capabilities.

Additional studies are needed to understand the effects of innate stimulation on mRNA-encoded HA vaccines, as well as to investigate the strain-specific differences observed between the vaccines. These differences could be due to variations in how efficiently HA proteins from different influenza strains are translated from the mRNA, their stability in maintaining a native conformation or their intrinsic immunogenicity. The influence of these factors may be critical to achieving comparable immune responses across different influenza strains and might require an optimized strain-specific dosing strategy in the vaccine formulation. Given the increasing risk of breakthrough infections as vaccine-induced antibody levels wane over time, it would be important to evaluate the durability of HA-specific antibody responses following mRNA immunization beyond the timeline of this study. Future research should also formally assess the development of cross-reactive antibody responses and map the specific HA epitopes targeted to better define the breadth and specificity of the humoral response. Furthermore, measuring HA-specific secretory IgA antibodies would provide valuable insights into the contribution of mucosal responses to vaccine-induced immunity. Although challenge experiments were outside the scope of this study, such models could provide valuable complementary data on protective efficacy in future work.

Whereas most approved mRNA vaccines incorporate nucleoside modifications to improve stability and reduce innate immune sensing^61^, the vaccine candidate tested in this study utilized unmodified mRNA. Nonetheless, there is evidence suggesting that despite differences in innate immune activation and dosing, the resulting adaptive responses elicited by the two vaccine modalities may be generally comparable, at least in some contexts^62^. In this study, the 160 µg dose of unmodified mRNA elicited stronger innate responses than those triggered by the licensed inactivated vaccines. This is consistent with the well-established capacity of mRNA platforms to activate innate immune sensors and drive early type I IFN-skewed responses, a feature observed across constructs, dose ranges and species^21,22,24,28,62^. However, we acknowledge that the dose used here is high and likely not tolerable in humans in a prophylactic setting, as it may trigger excessive early innate activation and exceed clinically acceptable reactogenicity thresholds. As nucleoside-modified mRNA constructs are generally associated with improved tolerability and are increasingly adopted, careful construct selection and dose optimization will be essential to achieve a favorable balance between immunogenicity and reactogenicity.

In summary, while all three vaccines induced virus-neutralizing and HAI antibodies (with some strain-dependent variation), the mRNA vaccine generated comparable or higher levels of HA-specific memory B cells and elicited greater T cell responses, along with a markedly stronger early innate immune response. These findings thus highlight the potential of the mRNA technology to drive robust adaptive immune responses, conceivably facilitated by a potent innate activation. Given its advantages in terms of lower production costs, rapid scalability and adaptability to circulating viral strains, the mRNA platform represents a promising alternative to traditional licensed influenza vaccines and holds considerable potential to strengthen seasonal influenza preparedness. Within this context, our findings in the non-human primate model offer important translational insights as influenza mRNA vaccines progress through late-stage clinical development.

The robust immunogenicity reported in Moderna’s phase 3 trial of mRNA-1010^20^ underscores the role of non-human primate models as a bridge for anticipating human immune responses. While our study employed an unmodified mRNA construct, it uniquely contributes to the field of mRNA vaccine development by providing mechanistic observations within lymphoid tissues. By examining these typically inaccessible compartments, such as the lymph nodes and spleen, we help elucidate the immunological processes underlying the vaccine’s immunogenicity beyond standard peripheral blood readouts. This offers a deeper level of immunological detail seldom captured in human clinical trials, where tissue sampling is inherently limited.

## Methods

### Vaccines

The experimental vaccine is a nucleoside-unmodified tetravalent mRNA formulation prepared by mixing four HA-coding mRNAs, representing influenza A/Michigan/45/2015 (H1N1)pdm09, A/Singapore/INFIMH-16-0019/2016 (H3N2), B/Phuket/3073/2013 and B/Colorado/06/2017, before encapsulation into a single LNP preparation. The mRNA constructs were produced by CureVac SE (Tübingen, Germany) based on their RNActive platform (claimed and described in patents WO2012019780 and US20150104476) and have a 5’ mCap structure (Cap 0), a GC-enriched open reading frame, parts of the 3’ untranslated region (UTR) of the *Homo sapiens* alpha-hemoglobin gene as the 3’ UTR, followed by a poly(A)_64_ stretch, a poly(C)_30_ stretch and a histone stem-loop^63^. The unmodified mRNA was encapsulated into LNPs composed of ionizable amino lipids, phospholipids, cholesterol and a PEGylated lipid using Acuitas Therapeutics’ technology (Vancouver, Canada)^63^. The licensed vaccines Vaxigrip (inactivated split-virion vaccine, manufactured by Sanofi Pasteur, 2018/2019 season)^2^ and Fluad (inactivated subunit vaccine adjuvanted with MF59, manufactured by Seqirus, 2018/2019 season)^3^ served as benchmark controls. The tetravalent form of Vaxigrip and the trivalent form of Fluad were used in this study. Vaxigrip contained the HAs of the same four influenza strains present in the mRNA vaccine, whereas Fluad did not include the B/Phuket/3073/2013 strain.

### Study design

Eighteen Chinese rhesus macaques (*Macaca mulatta*), nine males and nine females separated by gender, were housed in large cages at the Astrid Fagraeus Laboratory at Karolinska Institutet (Stockholm, Sweden). Animals were allocated into three study groups (*n* = 6 per group) with an even distribution of sex and body weight. Group 1 received the unmodified mRNA-LNP vaccine (160 µg), group 2 received Vaxigrip (full human dose, 60 µg) and group 3 received Fluad (full human dose, 45 µg). All animals were immunized by intramuscular injection at weeks 0 and 4 (prime-boost) and 12 of the 18 animals (*n* = 4 per group) were revaccinated after 8 months at weeks 35 and 39. Blood samples were collected at specific time points over the 41-weeks study period. Additionally, BAL fluid, spleen and lymph node samples were collected at study end. An illustration describing the study design is shown in Fig. 1A. For immunization and blood sampling, animals were sedated by intramuscular injection of a mild dose of ketamine. For BAL fluid sampling, animals were anesthetized with ketamine-xylazine or ketamine-medetomidine administered intramuscularly. At the end of the study, the animals that received four immunizations were euthanized under deep anesthesia induced with ketamine-xylazine or ketamine-medetomidine, followed by administration of an overdose of pentobarbital. Body weight, temperature, complete blood counts and clinical chemistry parameters were monitored throughout the study. Complete blood counts and clinical chemistry analysis were performed by Adlego Biomedical AB (now Scantox A/S; Solna, Sweden).

### Ethics

This study (18427-2019) was approved by the Stockholm Regional Ethical Board on Animal Experiments. All animal experiments were conducted following the guidelines and regulations of the Association for Assessment and Accreditation of Laboratory Animal Care and the Swedish Animal Welfare Agency.

### Sample processing

PBMCs were isolated from heparinized blood by standard density gradient centrifugation using Ficoll-Paque (GE Healthcare). PBMCs were either cryopreserved in heat-inactivated fetal calf serum (FCS) supplemented with 10% dimethyl sulfoxide (DMSO) or maintained in complete RPMI medium (RPMI 1640 medium supplemented with 10% FCS, 100 U/mL penicillin, 100 mg/mL streptomycin and 2 mM L-glutamine) at 37°C and 5% CO_2_, and used fresh in downstream applications. Cells from BAL fluid were separated by centrifugation and subsequent filtration through a 70 µm cell strainer. Spleen and lymph nodes were mechanically disrupted using a syringe plunger and filtered through a 70 µm cell strainer.

### Gene microarray

Blood samples at baseline and one day after the first immunization were collected for a gene microarray analysis by Karolinska Institutet’s Bioinformatics and Expression Analysis Core Facility (Huddinge, Sweden). Briefly, RNA extracted from whole blood was hybridized to Agilent Rhesus Macaque Gene Expression Microarrays version 2 (Agilent Technologies cat. no. G2519F-026806) for 17 h at 65°C in a rotating hybridization oven. After washing steps with GE wash buffer 1 and 37°C GE wash buffer 2 (Agilent Technologies), slides were immediately scanned using an Agilent DNA microarray scanner G2505C at 3 µm resolution (Agilent Technologies). Gene microarray data was transformed linearly and analyzed by quantile normalization of gProcessed Signals using the Feature Extraction software version 10.7.

### Transcriptomics data analysis

Raw expression data were log₂-transformed and filtered to retain probes with detectable expression above background in at least one sample. Probes with missing values were excluded. Gene IDs without an annotated gene symbol were retained as 5-digit numbers. Variance-stabilized fold change data (pre- vs. one day post-immunization) were subjected to a principal component analysis (PCA) using the *factoextra* package^64^ version 2.0.0 with unit variance scaling. Differential gene expression analysis was performed using the *limma* package^65^ version 3.65.1. For genes represented by multiple probes, the probe with the largest absolute moderated *t*-statistic was retained. Two separate models were fitted: (*i*) a paired model comparing pre- vs. one day post-immunization within each vaccine group, modeling animal ID as a blocking factor to account for repeated measures (Supplementary Data 1-3); (*ii*) an interaction model that included timepoint, vaccine group, and their interaction, to identify genes whose temporal expression changes differed by vaccine group (Supplementary Data 5). The first model was used to generate the volcano plots presented in Supplementary Fig. 1. The second model was used for the heat map visualization and subsequent module-level enrichment analysis presented in Fig. 1E.

Gene modules were defined by first scaling the expression matrix across genes, followed by hierarchical clustering using Euclidean distance and the Ward.D2 linkage method. The resulting dendrogram was cut to obtain six distinct gene modules, a number selected based on cluster stability and interpretability. False discovery rate (FDR) was controlled using the Storey-Tibshirani method^66^ to estimate *q*-values via the *qvalue* package^67^ version 2.42.0. Genes with *q*-value < 0.05 and an absolute log₂ fold change >1 were considered significantly differentially expressed. Enrichment was assessed by over-representation analysis (ORA) for Gene Ontology (GO) biological process terms, using significantly differentially expressed genes from the interaction model. A gene set enrichment analysis (GSEA) was performed using the previously described blood transcription modules^68^. ORA and GSEA were performed using the *clusterProfiler* package^69,70^ version 4.18.2. Terms with an FDR < 0.05 of a Fisher’s exact test were considered significant for the ORA. The GSEA was conducted on a ranked gene list based on log_2_ fold change and terms with an FDR < 0.1 were considered significant.

### Innate immunophenotyping

Immune cell subpopulations in PBMCs before and after vaccination were determined using flow cytometry. Freshly isolated PBMCs were stained with live/dead fixable blue viability dye (Invitrogen, cat. no. L23105) and FcR blocking reagent (Miltenyi, cat. no. 130-059-901) for 10 min at room temperature, followed by a panel of fluorescently labeled antibodies including anti-human CD3, CD4, CD14, CD16, CD19, CD20, CD66, IgG, IgM, IgD, HLA-DR, PD-1, CXCR5 and NKg2a (CD159a) for 20-30 min at 4°C (Table 1). Stained samples were washed with PBS and fixed using 1% paraformaldehyde (PFA) in PBS. Samples were acquired on a BD LSRFortessa flow cytometer (BD Biosciences) and the data were analyzed using FlowJo software (BD Life Sciences).

**Table 1:** Antibody panel used for innate immunophenotyping

Marker	Fluorochrome	Clone	Vendor	Cat. no.
CD3	APC-Cy7	SP34-2	BD Biosciences	557757
CD4	PE-Cy5.5	S3.5	Invitrogen	MHCD0418
CD14	BV510	M5E2	BioLegend	301842
CD16	AF700	3G8	BD Biosciences	560713
CD19	ECD	J3-119	Beckman Coulter	IM2708U
CD20	BV605	2H7	BioLegend	302334
CD66	APC	TET2	Miltenyi	130-118-539
IgG	BV421	G18-145	BD Biosciences	562581
IgM	PerCP-Cy5.5	G20-127	BD Biosciences	561285
IgD	FITC	Polyclonal	Southern Biotech	2030-02
HLA-DR	BV650	L243	BioLegend	307650
PD-1	BV785	EH12.2H7	BioLegend	329930
CXCR5	PE-Cy7	MU5UBEE	eBioscience	25-9185-42
NKg2a (CD159a)	PE	Z199	Beckman Coulter	IM3291U

### Plasma cytokines and chemokines

Cytokines and chemokines in plasma were analyzed by the Affinity Proteomics core facility, SciLifeLab (Stockholm, Sweden) using the ProcartaPlex NHP Cytokine & Chemokine Panel 30plex (Invitrogen) according to the manufacturer’s instructions. Samples were measured in a Luminex instrument. Curve-fitting and graphical representations were performed with Belysa Immunoassay Curve Fitting software (Millipore). Standard curves were generated using a 5-parameter logistic curve fit and absolute cytokine and chemokine concentrations were obtained by interpolation of the standard curve.

### ELISA

Half-area flat bottom 96-well plates were coated with 50 ng/well of either H1N1pdm09 (A/Michigan/45/2015), H3N2 (A/Singapore/INFIMH-16-0019/2016), B/Phuket/3073/2013 or B/Colorado/06/2017 HA protein (ProteoGenix) diluted in PBS and incubated overnight at 4°C. Plates were blocked with 5% milk powder in PBS (blocking buffer) for 1 h at room temperature. Plates were subsequently incubated with 5-fold serially diluted plasma (starting at 1:20) for 2 h at room temperature. HA-specific IgGs were detected after incubation for 1 h at room temperature with an HRP-conjugated goat anti-monkey IgG (Nordic MUBio, cat. no. 246-GAMon) diluted 1:20,000 in blocking buffer. Plates were washed three times with PBS containing 0.05% Tween 20 in between each step using an automatic plate washer. Signal was developed upon addition of TMB substrate solution (BioLegend, cat. no. 421101). The reaction was stopped after 7 min by addition of 1 M H_2_SO_4_. Absorbance was measured at 450 nm with background correction at 550 nm using an ELISA reader. Samples were analyzed in duplicate. Antibody titers are reported as half-maximal effective dilutions (ED_50_).

### Microneutralization assay

Neutralizing antibody titers were determined by VisMederi (Siena, Italy) using a microneutralization assay. Briefly, 2×10^3^ TCID_50_/mL of either A/Michigan/45/2015 (H1N1)pdm09, A/Singapore/INFIMH-16-0019/2016 (H3N2), B/Phuket/3073/2013 or B/Colorado/06/2017 influenza virus were pre-incubated with 2-fold serially diluted heat-inactivated serum (starting at 1:10) for 1 h at room temperature. Then, the serum/virus mixtures were transferred to 96-well plates containing Madin-Darby canine kidney (MDCK) cells and plates were incubated at 37°C and 5% CO_2_ for 3-5 days. Samples were analyzed in duplicate. Microneutralization titers are reported as the reciprocal of the highest dilution where no cytopathic effect (CPE) was observed.

### Hemagglutination inhibition assay

Functional antibody titers were measured in an HAI assay performed by VisMederi (Siena, Italy) according to the WHO standardized protocol. Briefly, 0.5% turkey red blood cells (Rockland Immunochemicals) were diluted in PBS. Receptor destroying enzymes (Denka Seiken) were used to avoid non-specific HAI during overnight incubation at 37°C. Serum samples were serially diluted 2-fold in V-bottom 96-well plates, starting from a 1:5 dilution. HA of either H1N1pdm09 (A/Michigan/45/2015), H3N2 (A/Singapore/INFIMH-16-0019/2016), B/Phuket/3073/2013 or B/Colorado/06/2017 influenza virus were added to diluted serum and incubated for 30 min at room temperature. Samples were analyzed in duplicate. The HAI titer was calculated by the reciprocal of the last serum dilution that resulted in non-agglutinated red blood cells.

### Antigen-specific memory B cells and germinal center B cells

PBMCs, spleen and lymph node cell suspensions were stained with live/dead fixable blue viability dye according to the manufacturer’s protocol (Invitrogen, cat. no. L23105). Subsequently, samples were stained with 100 ng of fluorescently labeled tetramer HA probes (H1N1pdm09 [A/Michigan/45/2015], H3N2 [A/Singapore/INFIMH-16-0019/2016], B/Phuket/3073/2013 and B/Colorado/06/2017) for 20-30 min at 4°C (Table 2). Tetramer HA probes were prepared by incubation of 4-fold molar excess of biotinylated HA protein with streptavidin (SA)-conjugated fluorophores. Then, cells were incubated with a panel of antibodies including anti-human CD3, CD14, CD16, CD19, CD20 and IgG for 20-30 min at 4°C (Table 2). Finally, cells were washed with PBS and fixed with TF fix/perm buffer (BD Biosciences) for 10 min at 37°C. Samples were acquired on an LSRFortessa flow cytometer (BD Biosciences) and the data analyzed with FlowJo software (BD Life Sciences). Germinal center B cells were detected following a similar procedure, with an additional step of intracellular staining using fluorescently labeled antibodies anti-human Bcl6 and Ki67 for 20 min at 4°C (Table 2).

**Table 2:** Probe and antibody panel used for detection of HA-specific memory B cells and germinal center B cells

Marker	Fluorochrome	Clone	Vendor	Cat. no.
A/H1/Michigan/15	SA-APC	N/A	BioLegend	405207
A/H3/Singapore/16	SA-BV421	N/A	BioLegend	405226
B/Phuket/13	SA-BV650	N/A	BioLegend	405231
B/Colorado/17	SA-AF488	N/A	BioLegend	405235
CD3	APC-Cy7	SP34-2	BD Biosciences	557757
CD14	APC-Cy7	M5E2	BioLegend	301820
CD16	APC-Cy7	3G8	BioLegend	302018
CD19	ECD	J3-119	Beckman Coulter	IM2708U
CD20	BV711	2H7	BioLegend	302342
IgG	BV786	G18-145	BD Biosciences	564230
Bcl6	PE-Cy7	K112-91	BD Biosciences	563582
Ki67	PE	B56	BD Biosciences	556027

### Recall antigen assay – in vitro T cell stimulation

HA-specific memory T cells in blood, BAL fluid and lymph nodes were analyzed based on intracellular cytokine production upon antigen re-stimulation as previously described^71^. Briefly, 1-2 million cells were cultured in complete RPMI medium at 37°C, 5% CO_2_ and were stimulated with 2 µg/mL of an HA overlapping peptide library comprising the four influenza strains (Thermo Scientific) in the presence of 10 µg/mL brefeldin A (Sigma-Aldrich). Stimulation with 1 µg/mL staphylococcal enterotoxin B (SEB) served as a positive control and stimulation with 0.04% DMSO in media served as a negative control. After overnight stimulation, cells were washed with PBS and stained with live/dead fixable blue viability dye (Invitrogen, cat. no. L23105) for 5 min at room temperature, followed by a panel of fluorescently labeled antibodies including anti-human CD4, CD8a, CD45RA and CCR7 for 20 min at room temperature (Table 3). Cells were fixed and permeabilized using the Cytofix/Cytoperm kit (BD Biosciences) and stained intracellularly with an additional panel of fluorescently labeled antibodies including anti-human CD3, CD69, IL-2, IL-13, IL-17A, IL-21, TNF and IFN-γ for 20 min at room temperature (Table 3). Cells were washed after staining and fixed with 1% PFA in PBS before acquisition on an LSRFortessa flow cytometer (BD Biosciences). Data was analyzed using FlowJo software (BD Life Sciences) and results were background subtracted using values from unstimulated cells.

**Table 3:** Antibody panel used for detection of HA-specific memory T cells

Marker	Fluorochrome	Clone	Vendor	Cat. no.
CD4	PE-Cy5.5	S3.5	Invitrogen	MHCD0418
CD8a	BV711	RPA-T8	BioLegend	301044
CD45RA	PE-Cy5	5H9	BD Biosciences	552888
CCR7	BV421	G043H7	BioLegend	353208
CD3	APC-Cy7	SP34-2	BD Biosciences	557757
CD69	ECD	TP1.55.3	Beckman Coulter	6607110
IL-2	BV605	MQ1-17H12	BD Biosciences	564165
IL-13	PE	JES10-5A2	BD Biosciences	559328
IL-17A	BV785	BL168	BioLegend	512338
IL-21	AF647	3A3-N2.1	BD Biosciences	562043
TNF	AF488	MAb11	BD Biosciences	557722
IFN-γ	AF700	B27	BioLegend	506516

### Data analysis and visualization

Prism 10 (GraphPad Software) was used to generate plots and perform statistical analysis, except for the transcriptomic data. Transcriptomic data were plotted and analyzed in R^72^ version 4.5.3, using the above-mentioned R packages. Statistical tests differed per analysis and are indicated in the description of each analysis and the corresponding figure legends. For comparison of the three vaccine groups, a Kruskal-Wallis test with Dunn’s multiple comparisons correction was used. Adjusted *p* values ≥ 0.05 were considered not significant.

## Supplementary information

Supplementary information

Supplementary information

Supplementary information

Supplementary information

Supplementary information

Supplementary information

## Data Availability

Transcriptomic data have been deposited at ArrayExpress under accession number E-MTAB-15632.

## Code availability

The code for the transcriptomic analysis is available on GitHub (https://github.com/Lore-Lab-Vaccine-Immunology/flu_vaccines_nhp).
