# Supplementary material for: Seasonal influenza mRNA vaccine induces stronger innate and comparable or better adaptive responses than licensed inactivated vaccines
Source: NPJ Vaccines. 2026 May 26;11:108. doi: 10.1038/s41541-026-01492-y (PMC13212880; doi:10.1038/s41541-026-01492-y)
Supplement: Supplementary file 1 — Supplementary information [file 41541_2026_1492_MOESM1_ESM.pdf]

Supplementary Information

Seasonal influenza mRNA vaccine induces stronger innate and comparable or better adaptive responses than licensed inactivated vaccines

Erick Bermúdez-Méndez[1,2], Klara Lenart[1,2,4], Rodrigo Arcoverde Cerveira[1,2], Olivia Engstrand[1,2], Fredrika Hellgren[1,2], Alberto Cagigi[1,2,5], Sebastian Ols[1,2,6], Edith Jasny[3], Annika Reinhardt[1,2], Benjamin Petsch[3,7], Karin Loré[1,2,*]

[1] Division of Immunology and Respiratory Medicine, Department of Medicine Solna, Karolinska Institutet and Karolinska University Hospital, Stockholm, Sweden

[2] Center for Molecular Medicine, Karolinska Institutet, Stockholm, Sweden

[3] CureVac SE, Tübingen, Germany

[4] Present address: Laboratory of Molecular Immunology, The Rockefeller University, New York, USA

[5] Present address: Europe Regional Office, International Vaccine Institute, Stockholm, Sweden

[6] Present address: Department of Biochemistry and Institute for Protein Design, University of Washington, Seattle, USA

[7] Present address: CiplaRNA GmbH, Reutlingen, Germany

* Corresponding author: karin.lore@ki.se

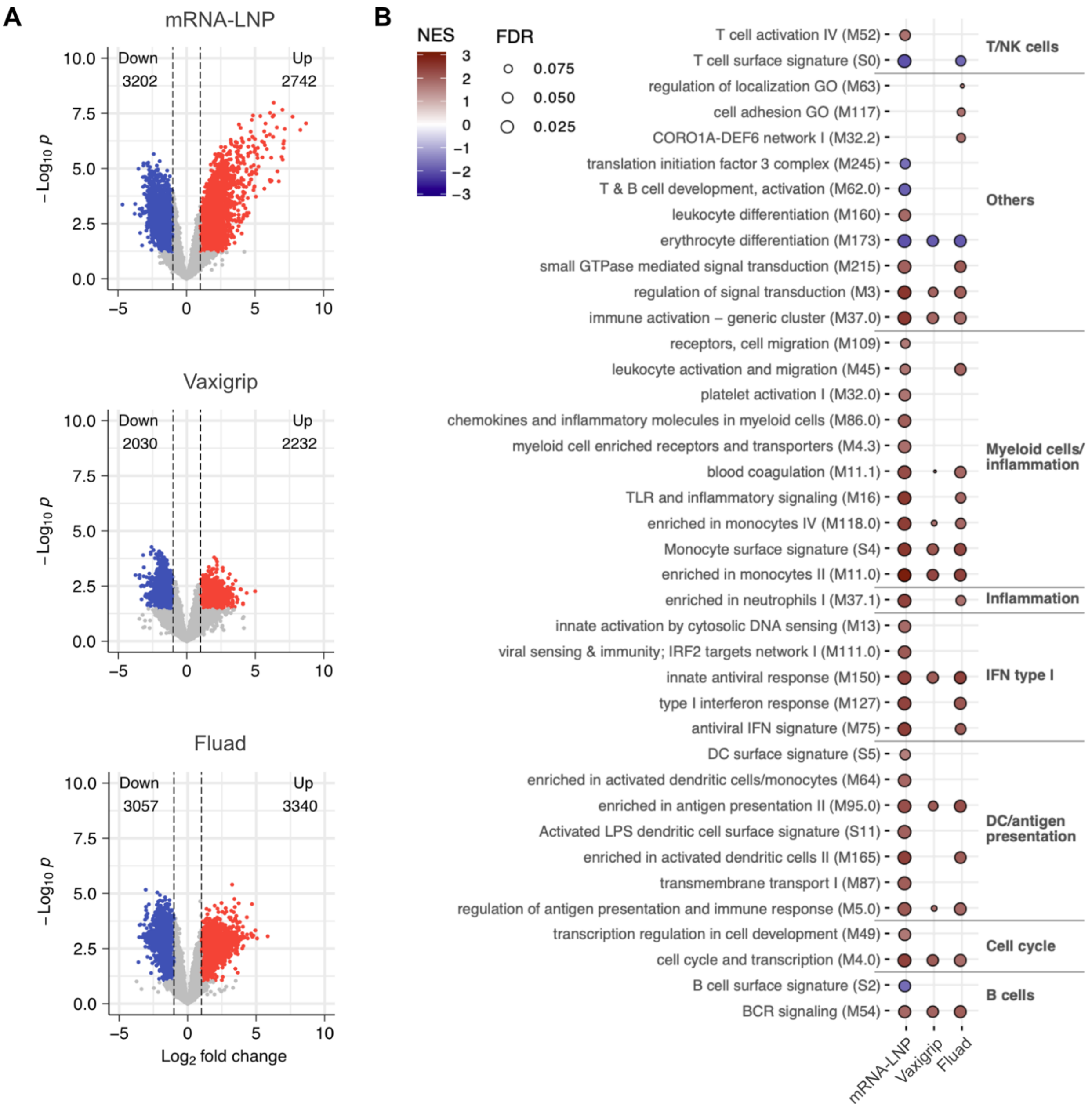

Supplementary Figure 1. Transcriptomic alterations in blood induced after vaccination.

A. Volcano plots of differential gene expression analysis in blood before and one day after the first immunization with either an unmodified mRNA-lipid nanoparticle (LNP) influenza vaccine, an inactivated split-virion vaccine (Vaxigrip) or an adjuvanted inactivated subunit vaccine (Fluad). Dots represent individual genes (n = 4-5 rhesus macaques per group). Genes with an absolute $\log_2$ fold change of 1 and a q-value <0.05 were considered significantly differentially expressed. The vertical dashed lines indicate the $\log_2$ fold change threshold. The numbers of significantly downregulated (blue) and upregulated (red) genes are indicated at the top corners of each plot.

B. Functional transcriptomic analysis. Top gene pathways significantly enriched among the three vaccine groups one day after the first immunization compared to baseline. The blood transcription modules were used as reference[1]. Dot color intensity depicts the normalized enrichment score (NES). Dot size represents false discovery rate (FDR) values.

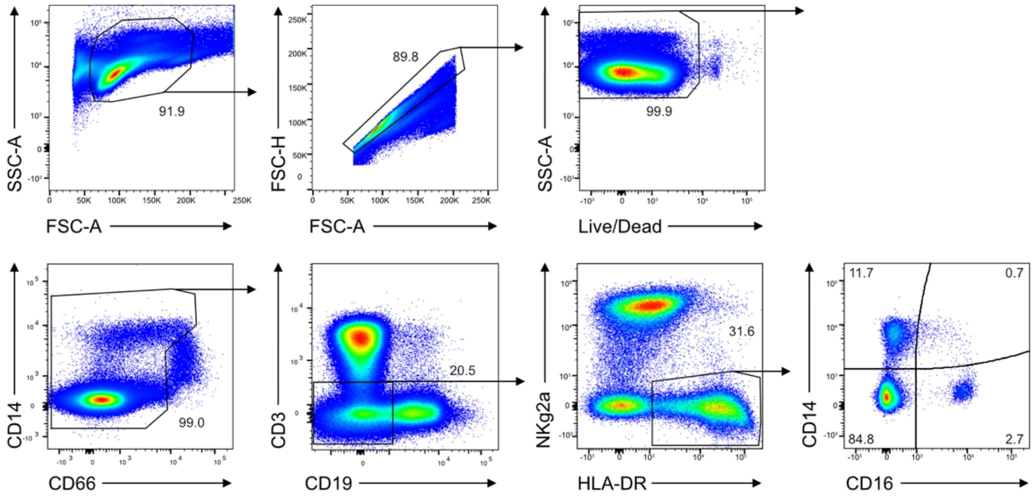

Supplementary Figure 2. Flow cytometry gating strategy for innate immunophenotyping.

Illustrative example of the gating strategy employed for the quantification of classical (CD14^{+}CD16^{-}), intermediate (CD14^{+}CD16^{+}) and non-classical monocytes (CD14^{-}CD16^{+}) in PBMCs before and after immunization. The panel of fluorescently labeled antibodies used for cell surface staining is detailed in **Table 1**. The plots depicted here correspond to an animal from the Vaxigrip group at one day after the first immunization.

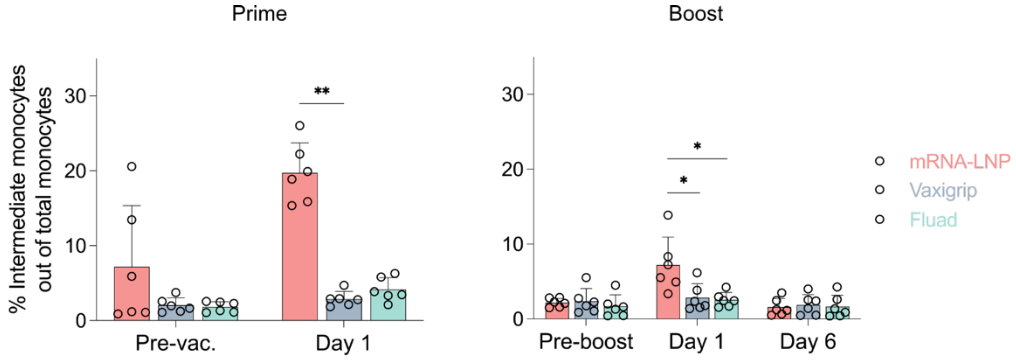

Supplementary Figure 3. Transient expansion of circulating CD14^{+}CD16^{+} intermediate monocytes following vaccination.

Relative frequency of CD14^{+}CD16^{+} intermediate monocytes among total monocytes before and one day after the first (left) and second (right) immunizations. Additionally, data at six days after the second immunization are shown. Dots represent data for individual animals (*n* = 6 per group). Bars show the group means and error bars indicate the standard deviation (SD). Significant statistical difference between the group medians was determined with a Kruskal-Wallis test with Dunn's multiple comparisons correction. *p <0.05, **p <0.01.

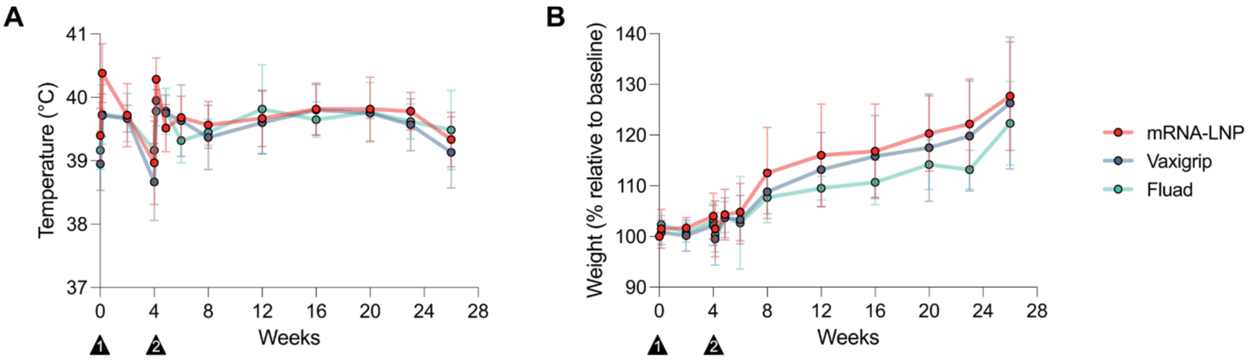

Supplementary Figure 4. Body temperature and weight monitoring following vaccination.
A. Body temperature of rhesus macaques at baseline (week 0) and the indicated time points (up to study week 26) following two doses (weeks 0 and 4) of either an mRNA-LNP vaccine, Vaxigrip or Fluad.
B. Body weight of rhesus macaques at the indicated time points (from study start and up to study week 26) following two doses (weeks 0 and 4) of the respective vaccines, expressed as percentage of weight relative to the baseline. Dots represent the group means (n = 6 rhesus macaques per group) at each timepoint and error bars indicate the SD. Black triangles at the bottom indicate the timing of the first and second immunizations.

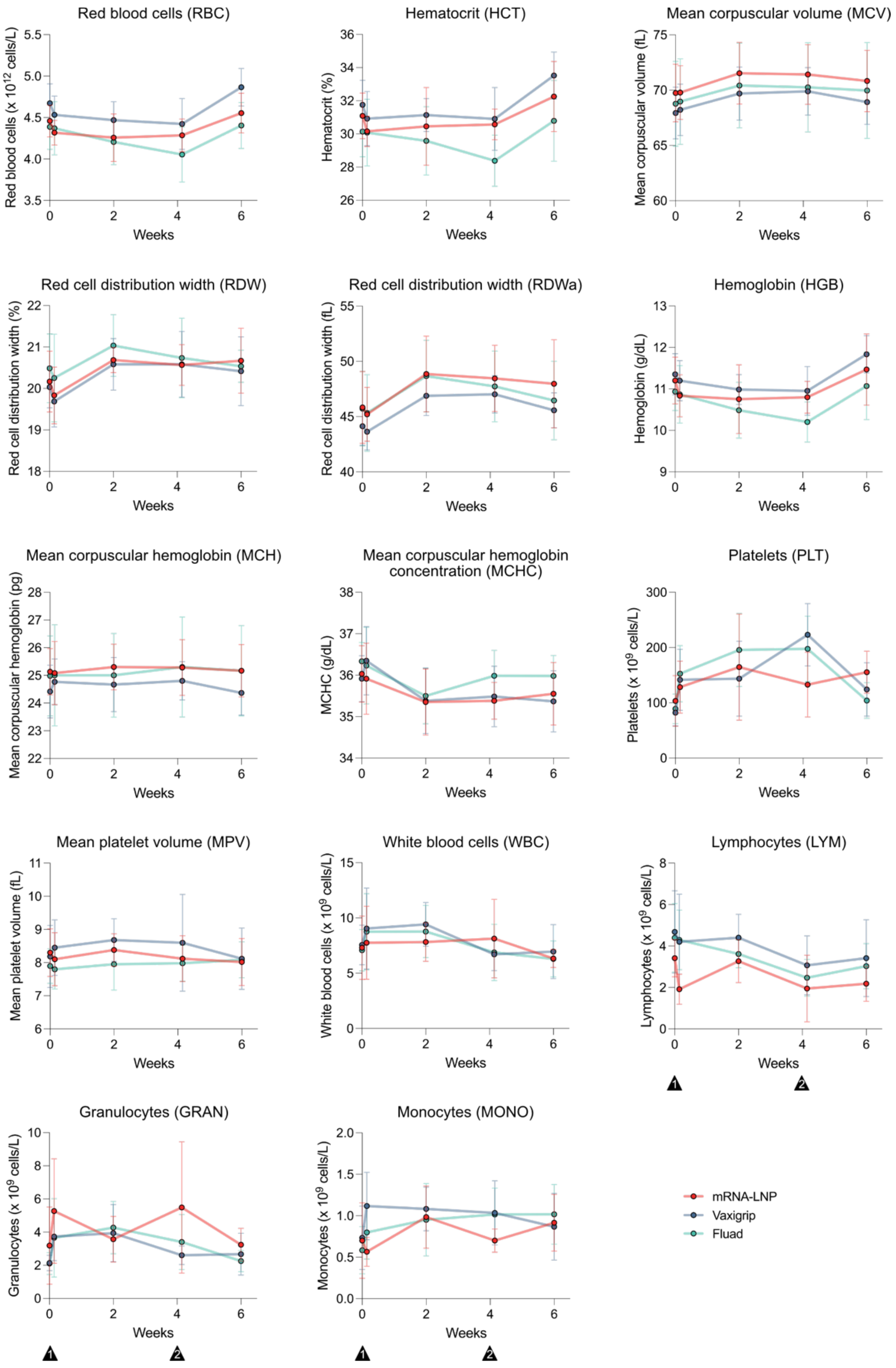

Supplementary Figure 5. Hematological parameters monitoring following vaccination.
Common hematological parameters in rhesus macaques at baseline (week 0) and the indicated time points (up to study week 6) following two doses (weeks 0 and 4) of either an mRNA-LNP vaccine, Vaxigrip or Fluad. Dots represent the group means (*n* = 6 per group) at each timepoint and error bars indicate the SD. Black triangles at the bottom indicate the timing of the first and second immunizations.

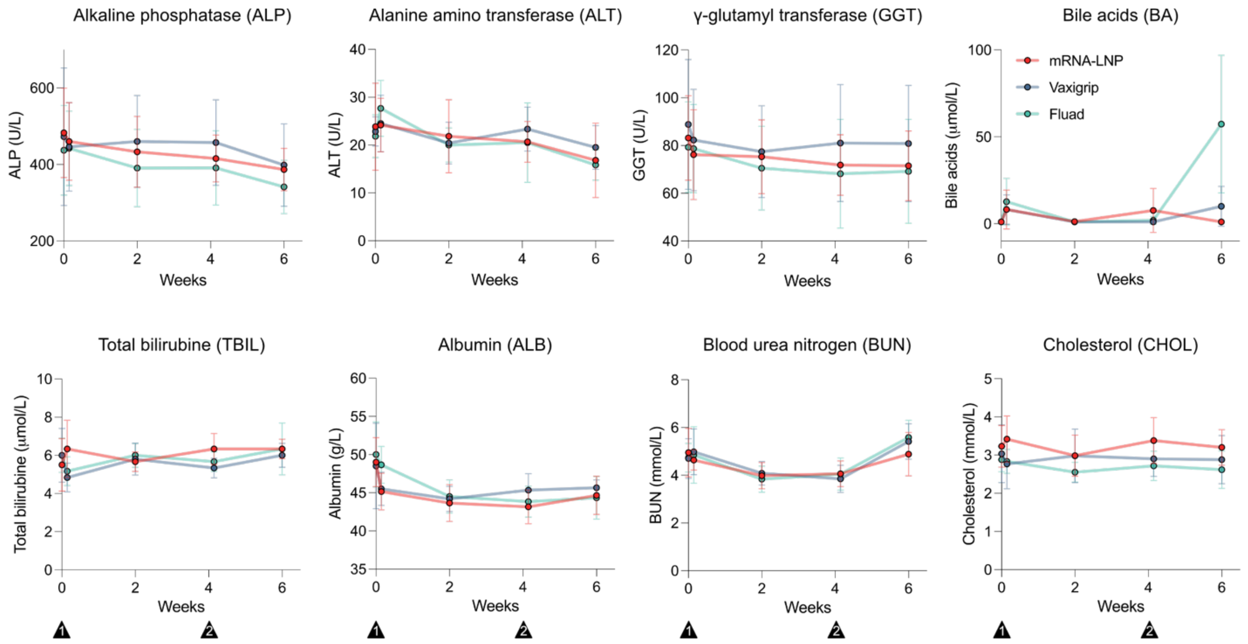

Supplementary Figure 6. Clinical chemistry parameters monitoring following vaccination.
Common clinical chemistry parameters in rhesus macaques at baseline (week 0) and the indicated time points (up to study week 6) following two doses (weeks 0 and 4) of either an mRNA-LNP vaccine, Vaxigrip or Fluad. Dots represent the group means (*n* = 6 per group) at each timepoint and error bars indicate the SD. Black triangles at the bottom indicate the timing of the first and second immunizations.

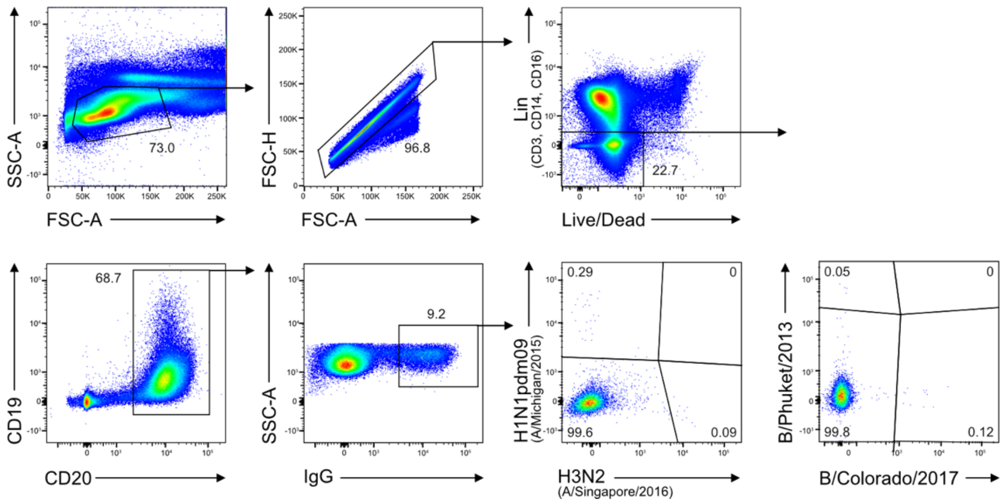

Supplementary Figure 7. Flow cytometry gating strategy for the quantification of HA-specific memory B cells.
Illustrative example of the gating strategy employed for the detection of HA-specific memory B cells in PBMCs, spleen and lymph node cell suspensions before and after immunization. The panel of fluorescently labeled antibodies used for cell surface staining is detailed in **Table 2**, which also included tetramer HA probes of four influenza strains (H1N1pdm09 [A/Michigan/45/2015], H3N2 [A/Singapore/INFIMH-16-0019/2016], B/Phuket/3073/2013 and B/Colorado/06/2017). The plots depicted here correspond to a PBMC sample from an animal in the mRNA-LNP group at week 8.

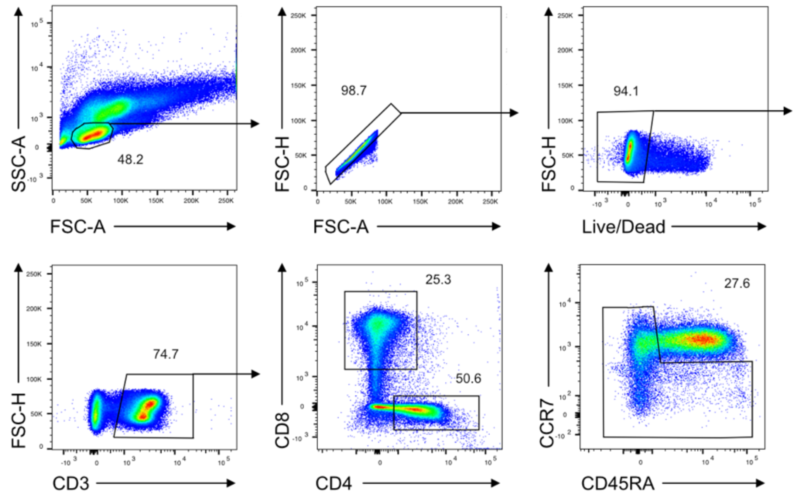

Supplementary Figure 8. Flow cytometry gating strategy for the quantification of memory T cells. Illustrative example of the gating strategy employed for the detection of $CD4^+$ and $CD8^+$ memory T cells in PBMCs, BAL fluid and lymph nodes at study week 41 (two weeks after the fourth immunization). This gating strategy precedes the quantification of HA-specific memory T cells shown in **Fig. 4D**. The panel of fluorescently labeled antibodies used for intracellular and cell surface staining is detailed in **Table 3**. The plots depicted here correspond to a PBMC sample from an animal in the mRNA-LNP group at week 41.

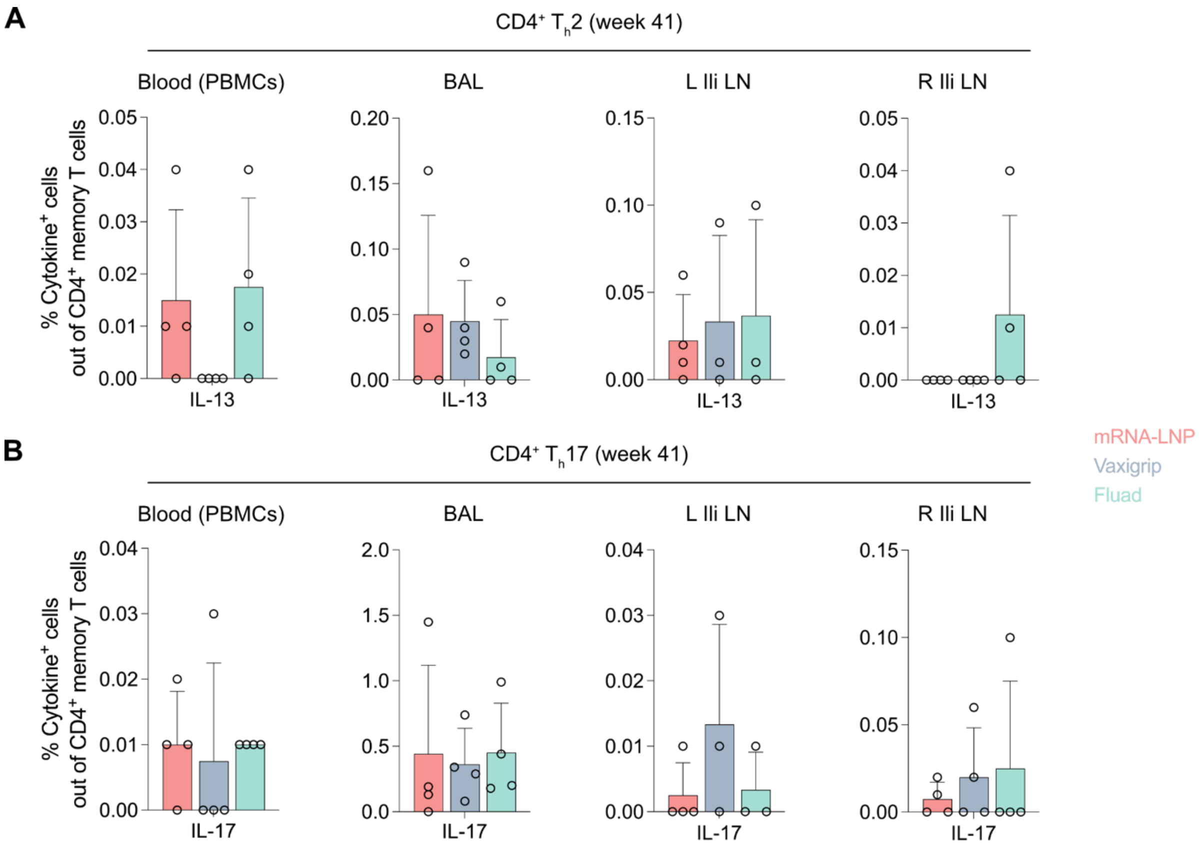

Supplementary Figure 9. Vaccine-induced HA-specific T_h2 and T_h17 cell responses.
A, **B.** Relative frequency of HA-specific IL-13 (**A**) and IL-17-producing (**B**) CD4⁺ T cells among total CD4⁺ memory T cells in blood, BAL fluid and lymph nodes at week 41 (two weeks after the fourth immunization) upon *in vitro* re-stimulation with overlapping HA peptides. Results were background subtracted based on values from unstimulated cells. Dots represent data for individual animals (*n* = 3-4 per group). Bars show the group means and error bars indicate the SD. L, left; R, right; Ili, iliac; LN, lymph node.

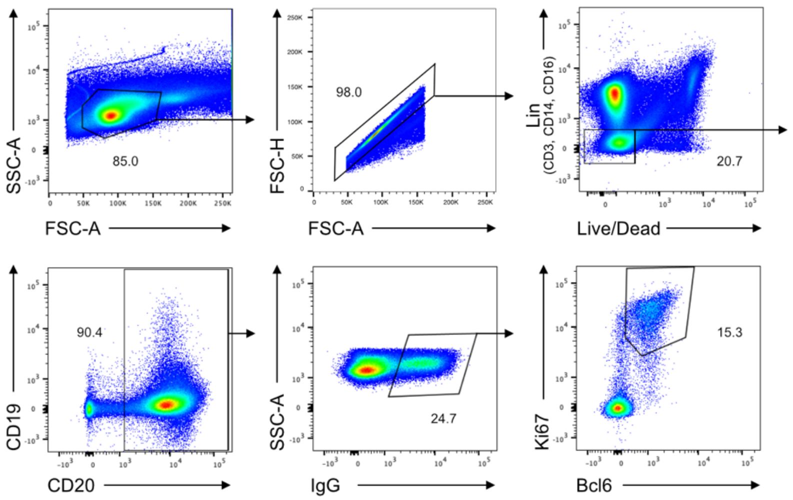

Supplementary Figure 10. Flow cytometry gating strategy for the quantification of germinal center B cells. Illustrative example of the gating strategy employed for the detection of germinal center B cells in the spleen and lymph nodes at week 41 (two weeks after the fourth immunization). The panel of fluorescently labeled antibodies used for cell surface staining is detailed in **Table 2**. The plots depicted here correspond to a right inguinal lymph node sample from an animal in the mRNA-LNP group.

Supplementary Data 1. Differentially expressed genes following immunization with the HA-coding tetravalent unmodified mRNA-LNP vaccine (one day post-immunization vs. baseline comparison) (Excel file).

Supplementary Data 2. Differentially expressed genes following immunization with Vaxigrip (one day post-immunization vs. baseline comparison) (Excel file).

Supplementary Data 3. Differentially expressed genes following immunization with Fluad (one day post-immunization vs. baseline comparison) (Excel file).

Supplementary Data 4. Unique and shared differentially expressed genes among the three vaccine groups following immunization (one day post-immunization vs. baseline comparison) (Excel file).

Supplementary Data 5. Differentially expressed genes from an interaction model that included timepoint, vaccine group, and their interaction, to identify genes whose temporal expression changes differed by vaccine (Excel file).

Supplementary References

1. Li, S. *et al.* Molecular signatures of antibody responses derived from a systems biology study of five human vaccines. *Nature Immunology* **15**, 195–204 (2014).
